# Determinants of neonatal sepsis among neonates in the northwest part of Ethiopia: case-control study

**DOI:** 10.1186/s13052-019-0739-2

**Published:** 2019-11-28

**Authors:** Mulunesh Alemu, Mulatu Ayana, Hailemariam Abiy, Biniam Minuye, Wubet Alebachew, Aklilu Endalamaw

**Affiliations:** 1 School of Public health, College of Health Sciences, Debre Markos University, Debre Markos, Ethiopia; 2 Department of Nursing, College of Health Sciences, Debre Tabor University, Debre Tabor, Ethiopia; 3 Department of Pediatrics and Child Health Nursing, School of Health Sciences, College of Medicine and Health Sciences, Bahir Dar University, Bahir Dar, Ethiopia

**Keywords:** Neonate, Sepsis, Ethiopia

## Abstract

**Background:**

Neonatal sepsis is one of the leading causes of neonatal morbidity and mortality. Despite implementing of different preventive interventions, the burden of neonatal sepsis is reporting in different areas of Ethiopia. For further interventions, identifying its determinants is found to be crucial.

**Objective:**

This study aimed to identify determinants of neonatal sepsis in the Northwest part of Ethiopia.

**Methods:**

Unmatched case-control study was conducted among 246 neonates admitted in neonatal intensive care unit, Northwest Ethiopia. Study participants were selected from February 1st to March 30th 2018. Data was collected through face to face interview and review of neonates’ medical records using pretested structured questionnaire. Data was entered into Epi Data version 4.2.0.0 and further transferred to SPSS statistical software version 25 for analysis. All independent variables with *p*-value < 0.25 in Bivariable analysis were entered into multivariable logistic regression analysis. Finally, variables with p-value < 0.05 were considered as determinants of neonatal sepsis.

**Results:**

A total of 82 cases and 164 controls were included in this study. Neonates with gestational age < 37 weeks [AOR = 6.90; 95% CI (2.76, 17.28)], premature rupture of membrane [AOR = 2.81; 95% CI (1.01, 7.79)], not crying immediately at birth and have received resuscitation at birth [AOR = 2.85; 95% CI (1.09, 7.47)] were found to be predictors of neonatal sepsis.

**Conclusions and recommendations:**

Premature rupture of membrane was found to be obstetric-related determinant of neonatal sepsis. Gestational age < 37 weeks, not crying immediately at birth, and have received resuscitation at birth were found to be neonatal-related risk factors of neonatal sepsis. Infection prevention strategies need to be strengthening and/or implementing by providing especial attention for the specified determinants.

## Background

Globally, each year over 2.5 million neonates died within the first month of life in 2017. It accounts for two-thirds of deaths of infants and nearly two-fifths of all deaths in under-five children. Most of the neonatal deaths occur in low-and middle-income countries [1]. Neonatal sepsis is a major cause of neonatal morbidity and mortality in developing countries [2]. It is highly prevalent in sub-Saharan Africa, south Asia, and Latin America with case fatality risk of 9.8% in the first month of life [3]. Septicemia, meningitis, pneumonia, arthritis, osteomyelitis, and urinary tract infections [4, 5] are some of the prior causes of sepsis.

Improving maternal health and nutrition [6], maternal immunization [7], clean delivery, intra-partum antibiotic prophylaxis, intravenous central line management, clean umbilical cord cutting, early detection and treatment of infectious disease, closed medication system [8–10], improvements perinatal care services, and producing trained health professionals, like skill birth attendants and neonatal nurses [11, 12] are implementing approaches to prevent neonatal sepsis.

In spite of those approaches, premature rupture of membrane (PROM), medical and surgical problems of mothers [13–15], history of maternal urinary tract infection or sexually transmitted infection, presence of intra-partum fever, gestational age < 37 weeks, APGAR score < 7 at 5th minute, need for artificial ventilation, not crying immediately at birth [16, 17], delay in care seeking, and lack of access to well-trained health workers [18, 19] are identified to be contributing factors of neonatal sepsis from previous studies. In the involvement of different determinants, the prevalence of neonatal sepsis from primary studies is reported frequently in different countries, including Ethiopia. Notably, in Shashemene Ethiopia showed 77.9% [20]; 29.3% in Kenya [21], 24% in Dar es SalaamTanzania [22], and 35.1% in Mandya, Karnataka, India [23].

To prevent further this neonatal problem and to achieve sustainable development goal by reducing neonatal mortality, data from different geographical area of Ethiopia is required. Therefore, identifying the predictors of neonatal sepsis is indicated which is helpful in designing strategies to prevent and/or treat neonatal sepsis.

## Objective

This study aimed to identify determinants of neonatal sepsis in the Northwest part of Ethiopia.

## Methods

### Study design, period, setting, and population

An institution based unmatched case-control study was conducted at Debre Markos Referral Hospital, Northwest Ethiopia from February 1st to March 30th 2018. The town is 299 km far from Addis Ababa, capital city of Ethiopia. This hospital has more than 193 bed capacity and it serves to more than 2,153,937 populations in its catchment area in the Northwest part of Ethiopia. All neonates who were admitted in neonatal intensive care unit (NICU) were the source population. Besides, all neonates who were admitted in NICU during the study period were the study population. Mothers who had hearing impairments or unable to talk, abandoned neonates who were admitted in the hospital, and neonates whose cards had incomplete information were excluded from the study in both controls and cases.

### Variables

**Dependent variable:** Neonatal sepsis.

**Independent Variables:** Socio-demographic characteristics of neonates and parents (age of the neonate, sex of the neonate, religion, ethnicity, maternal age, marital status of the parents, family educational status, family occupation, family size, and place of residence), maternal factors (gravidity, parity, complication during pregnancy and delivery, place of delivery, mode of delivery, PROM, duration of labour, and ANC follow-up) and neonatal factors (birth weight, gestational age, birth asphyxia, APGAR score) were the hypothesized determinants.

### Operational definitions

**Case (Neonatal sepsis):** The established Integrated Management of Neonatal and Childhood Illness (IMNCI) clinical features, includes the presence of two or more of persistent fever (≥37.5 °C) or persistent hypothermia (≤35.5 °C) for more than one hour, fast breathing (≥60 breath per minute), severe chest in drawing, grunting, not feeding well, movement only when stimulated, bulged fontanel, convulsion, lethargic or unconsciousness along with ≥2 of the hematological criteria such as total leukocyte count (< 4000 or > 12,000 cells/mm3), absolute neutrophil count (< 1500 cells/mm3 or > 7500 cells/mm3), platelet count (< 150 or > 450 cells/mm3), and random blood sugar (< 40 mg/dl or > 125 mg/dl) were used to diagnose neonatal sepsis cases.

**Control:** neonates with the diagnosis of non-sepsis case with their index mother.

### Sample size determination and sampling procedure

Sample size was determined using the Epi Info Version 3.5.1 software with the following assumptions: Two-sided confidence level (CI) =95%, Power = 80%, Ratio of controls to cases = 2:1, and from a case-control study conducted on risk factors for neonatal sepsis in public hospitals, Northern Ethiopia [16]. Then, the final sample size was 246 (82 Cases and 164 Controls).

Both cases (diagnosed with sepsis) and controls (not diagnosed with sepsis) were selected through consecutive sampling technique from the neonates who were admitted in NICU until the required sample size was reached.

### Data collection method and procedure

Data were collected through interviewing mothers and reviewing neonates’ medical records using checklist and structured data abstraction sheet. The abstraction sheet includes maternal sociodemographic, maternal obstetric and gynecological, prenatal, intranatal, and neonatal variables. The data was from February 1st to March 30th 2018. The data was collected by 3 BSc nurses and 1 BSc nurse supervisor.

### Data quality control

Pretest was done on 5% of the sample size. Training was given for data collectors and supervisor about data collection tool and data collection procedure. Data collection tool was prepared in English and translated to the national language (Amharic) and then back to English. Completeness of each abstraction sheet had been checked by the principal investigator and the supervisor in a daily base. Double data entry was done by two data clerks and consistency of the entered data was cross-checked.

### Data processing and analysis

Data was coded and entered into Epi-data Version 4.2.0.0 and then exported into SPSS version 25 for analysis. Descriptive statistics were carried out in texts and tables. Bivariable analysis was done and all variables with *p*-value < 0.25 were entered into the multivariable logistic regression. Variables with p-value < 0.05 were considered as determinants of neonatal sepsis.

### Ethical considerations

The study protocol was approved by research ethics and approval committee of Debre Markos University Health Science College. Official letter was written to Debre Markos Referral Hospital. Respondents/guardians had got information on the purpose of the study, its procedures, and their right to refuse or decline participation in the study at any time. Both written and verbal consent were obtained from the study participants/guardians. Confidentiality was also assured.

## Results

### Socio-demographic characteristics of the mothers and neonates

A total of 82 neonates who had sepsis (cases) with their index mothers and 164 neonates who had no sepsis (controls) with their index mothers were included. The median age of mothers of neonates with sepsis was 25 years and 28 years for controls. More than half (58.5%) of cases and 39% controls were living in rural area. Regarding to marital status, 95.1% cases’ mothers and 98.2% of controls’ mothers were married. Nearly half (48.8%) of cases’ and 40.2% of controls’ mothers were farmers by occupation and 48.8% cases’ and 38.4% of controls’ mothers had not attended formal education. About neonates’ socio-demographic characteristics, 90.2% of the cases and 92.7% controls were found under the age of 07 days and the median age at admission was 12 h for both cases and controls. The proportions of male neonates were 43.9% in cases and 40.9% in controls. Almost all (99.2%) of respondents were Amhara by their ethnic group (Table 1).

**Table 1:** Socio-Demographic Characteristics of cases and controls attending Neonatal intensive care unit in the Northwest part of Ethiopia, 2018

Variables	Category	Case *n* = 82(%)	Control *n* = 164(%)	Total *n* = 246(%)
Age of mother	15–19	1(1.2)	1(0.6)	2(0.8)
	20–24	27(32.9)	33(20.1)	60(24.4)
	25–29	29(35.4)	64(39)	93(37.8)
	30–34	11(13.4)	47(28.7)	58(23.6)
	35–39	6(7.3)	16(9.8)	22(8.9)
	> 40	8(9.8)	3(1.8)	11(11.6)
Marital status	Single/divorced/separated	4(4.6)	3(1.8)	7(2.8)
	married	78(95.1)	161(98.2)	239(97.2)
Religion	Orthodox	81(98.8)	143(87.2)	224(91.1)
	Muslim	1(1.2)	18(11)	19(7.7)
	Protestant	0(0)	3(1.8)	3(1.2)
Residence	Urban	34(41.5)	100(61)	134(54.5)
	Rural	48(58.5)	64(39)	112(45.5)
educational level of the mother	No formal education	40(48.8)	63(38.4)	103(41.9)
	Primary	23(28)	46(28,1)	69(28)
	Secondary	11(13.4)	35(21.3)	46(18.7)
	college and above	8(9.8)	20(12.2)	28(11.4)
educational level of the father	No formal education	40(48.8)	56(34.1)	96(39)
	Primary	21(25.6)	34(20.7)	55(22.4)
	Secondary	10(12.2)	37(22.6)	47(19.1)
	college and above	7(8.5)	34(20.7)	41(16.7)
occupation of the mother	Farmer	40(48.8)	66(40.2)	106(43.1)
	House wife	31(37.8)	65(39.6)	96(39)
	Government employee	7(8.5)	20(12.2)	27(11)
	Merchant	4(4.9)	13(7.9)	17(6.9)
Occupation of the father	Farmer	45(54.9)	65(39.6)	110(44.7)
	daily labor	12(14.6)	33(20.1)	45(18.3)
	government employee	8(9.8)	38(23.2)	46(18.7)
	Merchant	13(15.9)	24(15.2)	38(15.4)
number of family members	< 5	67(81.7)	139(84.8)	206(83.7)
	> = 5	15(18.3)	25(15.2)	40(16.3)
age of neonate in days	< 7	74(90.2)	152(92.7)	226(91.9)
	7–28	8(9.8)	12(7.3)	20(8.1)
sex of the neonate	Male	36(43.9)	67(40.9)	103(41.9)
	Female	46(56.1)	97(59.1)	143(58.1)

### Maternal-related variables

The median parity of mothers for both cases and controls was 2 and ranges from 1 to 9 live births for cases and 1 to 6 for controls. Majority of the respondents (80.5%) cases and 89.6% controls had single pregnancy status at the index pregnancy. Most of the study participants (96.7%) have received antenatal care (ANC) service at least once during the index pregnancy. More than two thirds (70.7%) of cases and 79.9% controls were delivered by spontaneous vaginal delivery. The proportion of mothers who had history of urinary tract infections (UTI) during the index pregnancy were 19.5% in cases and 2.4% in controls. Nearly one-third (35.4%) of cases’ mother gave birth after 18 h of rupture of membrane (PROM) and 8.5% in controls group (Table 2).

**Table 2:** Maternal factors of cases and controls attending Neonatal intensive care unit in the Northwest part of Ethiopia, 2018

Variables	Category	Case *n* = 82(%)	Control *n* = 164(%)	Total *n* = 246(%)
Gravidity	< 5	73(89)	160(97.6)	233(94.7)
	≥5	9(11)	4(2.4)	13(5.3)
Parity	< 5	75(91.5)	160(97.6)	235(95.5)
	≥5	7(8.5)	4(2.4)	11(4.5)
current pregnancy status	Single	66(80.5)	147(89.6)	213(86.6)
	Multiple	16(19.5)	17(10.4)	33(13.4)
ANC follow up	Yes	74(90.2)	164(100)	238(96.7)
	No	8(9.8)	0(0)	8(3.3)
number of ANC visit	< 3	23(28)	27(16.5)	50(20.3)
	≥3	51(62.2)	137(83.5)	188(76.4)
APH	Yes	5(6.1)	0(0)	5(2)
	No	77(93.9)	164(100)	237(98)
PIH	Yes	2(2.4)	7(4.3)	9(3.7)
	No	80(97.6)	157(95.7)	237(96.3)
GDM	Yes	82(100)	1(0.6)	1(0.4)
	No	0(0)	163(99.4)	245(99.6)
UTI	Yes	16(19.5)	4(2.4)	20(8.1)
	No	66(80.5)	160(97.6)	226(91.9)
Mode of delivery	SVD	58(70.7)	131(79.9)	189(76.8)
	CS	9(11)	18(11)	27(11)
	assisted/instrumental	15(18.3)	15(9.1)	30(12.2)
Place of delivery	Hospital	47(57.3)	118(72)	165(67.1)
	health center	32(39)	46(28)	78(31.7)
	clinics/home	3(3.6)	0(0)	3(1.2)
Duration of labour	< 6	21(25.6)	43(26.8)	65(26.4)
	6–12	26(31.7)	75(45.7)	101(41.1)
	12–24	29(35.4)	45(27.4)	74(30.1)
	≥24	6(7.3)	0(0)	6(2.4)
PPH	Yes	3(3.7)	0(0)	3(1.2)
	No	79(96.3)	164(100)	243(98.8)
Mal presentation	Yes	2(2,4)	1(0.6)	3(1.2)
	No	80(97.6)	163(99.4)	243(98.8)
PROM	< 18 h	53(64.63%)	150(91.5%)	203 (82.5%)
	≥18 h	29(35.37%)	14(8.5%)	43 (17.5%)

*APH* ante partum hemorrhage, *PPH* postpartum hemorrhage, *GDM* gestational diabetes mellitus, *PIH* pregnancy induced hypertension, *PROM* premature rupture of membrane

### Neonatal-related variables

Two-third (67.1%) of controls and 29.3% of cases were term neonates. Majorities (62.2%) of cases and 37.1% of controls were born with below normal birth weight. More than half (53.7%) of cases and only 5.5% controls had APGAR score of < 7 at fifth minute. Besides, 53.7% of cases and 10.4% of controls have received resuscitation at birth (Table 3).

**Table 3:** Neonatal factors of cases and controls attending Neonatal intensive care unit in the Northwest part of Ethiopia, 2018

Variables	Category	Case *n* = 82(%)	Control *n* = 164(%)	Total *n* = 246(%)
Cry immediately at birth	Yes	38(46.3)	147(89.6)	185(75.2)
	No	44(53.7)	17(10.4)	61(24.8)
Resuscitation done	Yes	44(53.7)	17(10.4)	61(24.8)
	No	38(46.3)	147(89.6)	185(75.2)
APGAR score in the first minute	< 7	34(41.5)	26(15.9)	60(24.4)
	≥7	48(58.5)	138(84.1)	186(75.6)
APGAR score in the fifth minute	< 7	44(53.7)	9(5.5)	53(21.5)
	≥7	38(46.3)	155(94.5)	193(78.5)
Birth weight	< 2500	51(62.2)	61(37.1)	112(45.5)
	2500–3999	30(36.6)	100(61)	130(52.8)
	≥4000	1(1.2)	3(1.9)	4(1.6)
Gestational age	< 37 weeks	50 (60.98%)	40 (24.39%)	90 (36.6%)
	37–42 weeks	24 (29.27%)	110 (67.07%)	134 (54.5%)
	≥42 weeks	8 (9.76%)	14(8.54%)	22 (8.9%)

### Determinants of neonatal sepsis

In multivariable logistic regression analysis, preterm neonates (gestational age < 37 weeks) had 6.9 times [AOR = 6.903; 95% CI (2.79, 17.28)] higher odds of developing sepsis compared to term neonates. The odds of neonatal sepsis among neonates born from mothers who had history of PROM was 2.8 times [AOR =2.81; 95% CI (1.01, 7.79)] as compared to their counterparts. Similarly, the odds of neonatal sepsis among neonates who were not crying immediately at birth and have received resuscitation at birth was 2.85 [AOR = 2.85; 95% CI (1.09, 7.47)] times higher compared to neonates who were crying immediately and did not received resuscitation at birth (Table 4).

**Table 4:** Bivariable and multivariable logistic regression analysis result of cases and controls attending Neonatal intensive care unit in the Northwest part of Ethiopia, 2018

Variables	Category	Cases n = 82(%)	Control n = 164(%)	COR [95%CI]	AOR [95%CI]	p-value
Residence	Urban	34(41.5)	100(61)	1	1	
	Rural	48(58.5)	64(39)	2.206[1.286,3.785]	0.612[0.142,2.641]	0.510
Educational status of the father	No formal education	40(48.8)	56(34.1)	3.469[1.398,8.611]	3.238[0.849,12.347]	0.085
	primary	21(25.6)	34(20.7)	3.000[1.128,7.982]	3.961[0.937,16.744]	0.061
	Secondary	10(12.2)	37(22.6)	1.313[0.449,3836]	1.278[0.286,5.715]	0.74
	Collage and above	7(8.5)	34(20.7)	1	1	8
Gravidity	< 5	73(89)	160(97.6)	1	1	
	≥5	9(11)	4(2.4)	4.932[1.471,16.536]	0.544[0.039,7.614]	0.651
Parity	< 5	75(91.5)	160(97.6)	1	1	
	≥5	7(8.5)	4(2.4)	3.733[1.060,13.145]	1.670[0.226,12.335]	0.615
Number of ANC visit	< 3	23(28)	27(16.5)	2.288[1.204,4.350]	1.658[0.608,4.527]	0.323
	≥3	51(62.2)	137(83.5)	1	1	
UTI	Yes	16(19.5)	4(2.4)	9.697[3.124,30.095]	2.981[0.628,14.147]	0.169
	No	66(80.5)	160(97.6)	1	1	
PROM	Yes	29(35.4)	14(8.5)	5.863[2.881,11.93]	2.812[1.015,7.787]**	0.048
	No	53(64.6)	150(91.5)	1	1	
Mode of delivery	SVD	58(70.7)	131(79.9)	1	1	
	CS	9(11)	18(11)	1.129[0.479,2.663]	2.019[0.534,7.636]	0.301
	Instrumental assisted	15(18.3)	15(9.1)	2.258[1.036,4.925]	2.021[0.638,6.397]	0.232
Gestational age	< 37	50(61)	40(24.4)	5.729[3.124,10.507]	6.903[2.758,17.28]**	0.001
	37–42	24(29.3)	110(67.1)	1	1	
	≥42	8(9.8)	14(8.5)	2.619[0.988,6.940]	2.435[0.604,9.818]	0.211
APGAR score at first minute	< 7	34(41.5)	26(15.9)	3.760[2.049,6.899]	2.271[0.931,5.539]	0.071
	≥7	48(58.5)	138(84.1)	1	1	
APGAR score at fifth minute	< 7	44(53.7)	9(5.5)	19.942[8.96,44.38]	17.67[6.109,51.12]**	0.001
	≥7	38(35.4)	155(94.5)	1	1	
Cry immediately at birth	Yes	38(46.3)	147(89.6)	1	1	
	No	44(53.7)	17(10.4)	10.01[5.156,19.44]	2.853[1.089,7.474]**	0.033
Resustation	Yes	44(53.7)	17(10.4)	10.01[5.156,19.44]	2.853[1.089,7.474]**	0.033
	No	38(46.3)	147(89.6)	1		

** significant variables

## Discussion

The finding of this study showed that both maternal and neonatal-related factor had a significant effect on the risk of neonatal sepsis. The result of this study showed that history of maternal PROM, being preterm neonate, those neonate who are not crying immediately at birth and have received resuscitation at birth were predictors of neonatal sepsis.

This study revealed that preterm delivery as one of the significant risk factors of neonatal sepsis which is 6.9 times more likely as compared to term delivery. This finding is in agreement with the findings from the study conducted in referral hospitals of Addis Ababa Ethiopia, Ghana, and Tanzania [13, 15, 19, 24, 25]. Explained that preterm neonates tend to have poor host defenses and more likely to suffer sepsis. Moreover, preterm neonates are also more likely to receive parenteral nutrition through insertion of needle to vein, which might expose neonates to infections.

Not crying immediately at birth showed a significant association with neonatal sepsis. The present study indicated that the odds of neonatal sepsis among neonates who were not crying immediately at birth were 2.85 times more likely as compared to those who were crying at birth. This finding was in line with the previous finding reported in Ghana and Mekele, Ethiopia [13, 16]. This might be crying at birth is the most critical part of the physiological adjustment procedure for a newborn to survive the extra uterine life following cutting of the umbilical cord when the newborn undergoes a series of cardiopulmonary changes with the first breath of air. Initial breathing is the result of a reflex triggered by pressure and temperature changes, noise, light, and other sensations related to the birth process. Neonate might be unable to cry due to interference of respiration. The absence of breathing and/or crying might leads health care professionals to do resuscitation. Resuscitation at birth was significantly associated with neonatal sepsis. Doing resuscitation to a newborn is also found to be a significant determinant of neonatal sepsis in the current and in the previous studies in Ghana and Tanzania [13, 15]. This happened because the lumen of the peripheral airway of the newborn is narrow and respiratory secretions are plentiful which could predispose the newborn to atelectasis. For the collapsed of lungs, performing vigorous procedures is may cause bruises to the delicate and fragile mucous membrane of the neonate and further serve as an entry point for microbial agents. Moreover, resuscitation might be done with unsterile equipment, which could introduce microbes into the lungs of the neonate whose immune system is not yet well developed.

From obstetrics related variables, premature rupture of membrane (≥18 h) is found to be a significant determinants of neonatal sepsis. The odds of neonatal sepsis among neonates born from mothers who had history of PROM was 2.8 times more likely as compared to those neonates born before 18 h of rupture of membrane. This finding is similar with the findings in Mekelle, Ethiopia and Pakistan [16, 26]. However, the study in Ghana [13] did not identify premature rupture of membrane as a significant risk factor. The contradiction may be due to appropriate interventions put in place to manage such cases in Ghana. Early rupture of membrane increases the chance of ascending microorganisms from the birth canal into the amniotic sac causing Chorioamnionitis and fetal compromise as well as asphyxia which frequently leads the new born to infection in utero. Cervical incompetence, cord prolapse, and mal-presentation associated with prematurity could causes PROM and further neonatal sepsis is happened [27].

## Limitation of the study

Since the study was done on admitted neonates, thus results might lack generalizability to the entire population.

## Conclusions

PROM was found to be obstetric-related determinant of neonatal sepsis, Gestational age < 37 weeks, not crying immediately at birth, and have received resuscitation at birth were found to be neonatal-related risk factors of neonatal sepsis. Infection prevention strategies need to be strengthening and/or implementing by providing especial attention for the specified determinants.

## Data Availability

All the data are available in the manuscript.

## Abbreviations

ANC: Antenatal care
AOR: Adjusted odds ratio
APGAR: Activity, pulse, grimace, appearance, respiration
BW: Birth weight
CI: Confidence interval
EONS: Early Onset Neonatal Sepsis; Integrated Management of Neonatal and Childhood Illness
LONS: Late onset neonatal sepsis
NICU: Neonatal intensive care unit
OR: Crude odds ratio
PROM: Premature rupture of membrane
SPSS: Statistical package for social science
UTI: Urinary tract infection
